# SAR System for UAV Operation with Motion Error Compensation beyond the Resolution Cell

**DOI:** 10.3390/s8053384

**Published:** 2008-05-23

**Authors:** José-Tomás González-Partida, Pablo Almorox-González, Mateo Burgos-García, Blas-Pablo Dorta-Naranjo

**Affiliations:** 1 Universidad Politécnica de Madrid. Departamento de Señales, Sistemas y Radiocomunicaciones, Av. Complutense s/n, 28040 Madrid, Spain; E-mail: almorox@gmr.ssr.upm.es; 2 Universidad de Las Palmas de Gran Canaria. Departamento de Señales y Comunicaciones. Edificio de Electrónica y Telecomunicaciones, 35017 Campus Universitario de Tafira, Las Palmas de Gran Canaria, Spain; E-mail: pdorta@dsc.ulpgc.es

**Keywords:** Unmanned Aerial Vehicle (UAV), Synthetic Aperture Radar (SAR), motion compensation, range alignment, Phase Gradient Autofocus (PGA)

## Abstract

This paper presents an experimental Synthetic Aperture Radar (SAR) system that is under development in the Universidad Politécnica de Madrid. The system uses Linear Frequency Modulated Continuous Wave (LFM-CW) radar with a two antenna configuration for transmission and reception. The radar operates in the millimeter-wave band with a maximum transmitted bandwidth of 2 GHz. The proposed system is being developed for Unmanned Aerial Vehicle (UAV) operation. Motion errors in UAV operation can be critical. Therefore, this paper proposes a method for focusing SAR images with movement errors larger than the resolution cell. Typically, this problem is solved using two processing steps: first, coarse motion compensation based on the information provided by an Inertial Measuring Unit (IMU); and second, fine motion compensation for the residual errors within the resolution cell based on the received raw data. The proposed technique tries to focus the image without using data of an IMU. The method is based on a combination of the well known Phase Gradient Autofocus (PGA) for SAR imagery and typical algorithms for translational motion compensation on Inverse SAR (ISAR). This paper shows the first real experiments for obtaining high resolution SAR images using a car as a mobile platform for our radar.

## 1. Introduction

Synthetic Aperture Radars (SAR) typically provide a two-dimensional representation of the scatterers over an extensive area that has been illuminated with microwaves. These two dimensions are called slant range and azimuth resolution. It is known that SAR systems obtain high range resolution transmitting signals with a large bandwidth. For very high resolution SAR systems, the movement errors could be large enough to shift the detected target to other range cells due to non-ideal motion. These error corrections are critical for achieving the required quality of the final SAR product.

Focusing SAR images with movement errors beyond the resolution cell has been addressed in the specialized literature [1, 2]. Typically, proposed techniques are based on two-step procedures. The first step consists of coarse motion compensation with resolution cell accuracy. Information provided by an Inertial Measuring Unit (IMU) is usually available for this purpose. The second step consists of fine motion compensation based on the received raw data. This fine correction is usually based on Signal Based Motion Compensation (SBMC) techniques, and their mission is to carry out a phase correction for auto-focusing the images.

Currently, the miniaturization of SAR systems is a very important objective because there are many applications in which the weight and size of the system are critical, for example UAV operation. Our research group is developing a miniaturized system for this purpose. From the perspective of miniaturization, it is useful that the radar system operates at very high carrier frequency, that is, millimeter-wave band (Ka band, 34 GHz) [3]. Thus, circuits and antennas are smaller and MMIC technology has just been made available. Furthermore, a large bandwidth can be more easily transmitted working in the millimeter-wave band, because the relative bandwidth is lower. The SAR system described in this paper transmits to 2 GHz of bandwidth, which corresponds to range resolution of 7.5 cm [4]. This high resolution is a problem from the point of view of motion errors, because the motion errors could be larger than the resolution cell in UAV operation.

A potential solution to this problem consists of range aligning the target response for each pulse, thus removing the range shift. This solution is only feasible for far scenes with a narrow swath, that is, with a range curvature that is very similar for all the targets in the scene. This is not valid for nearer or wider scenes due to the range curvature. The range curvature is different for each target because it depends on its position at the scene. This range curvature is generated by the ideal motion of the platform. The Range Migration Algorithm (RMA) is known to be able to focus SAR images with high and different range curvatures within the swath of interest. However, RMA can not compensate for the range cell shift due to the non-ideal motion of the platform, which results in defocusing. An additional technique to correct these effects is needed.

Another solution is to use the motion information provided by an IMU to align the target response for each pulse, removing the range shift. However, the system might not have available data on this motion because the addition of an IMU increases the weight and size of the SAR system, which might not be tolerable for some UAVs. This paper describes a method for focusing SAR images with movement errors larger than the resolution cell, without using an IMU. This method is based on a combination of the well known Phase Gradient Autofocus (PGA) for SAR imagery [5, 6, 7], with typical algorithms for translational motion compensation on Inverse SAR (ISAR), such as Envelope Correlation (EC) [8, 9] and Global Range Alignment (GRA) [10].

The main characteristics of the proposed motion compensation method are summarized as follows:

- It does not require IMU or sensor movements.
- It requires prominent scatters within the swath to collect the phase error using PGA.
- It does not compensate the range curvature due to the ideal trajectory of the platform. Therefore, it can be used in combination with algorithms that focus SAR images with different range curvatures, e.g., RMA.
- It is valid for stripmap-mode SAR operation.

This paper is arranged as follows:

Section two describes the different subsystems of the radar that have been developed. Specific measurements of each subsystem are presented.

Section three describes the SAR signal processing chain, emphasizing the motion compensation algorithm. Simulated data are presented to shown the performance of this technique for UAV applications.

The last section shows two real SAR images using the proposed system in a ground SAR system with a car as the mobile platform. These are the first experiments to prove the feasibility of our radar in a SAR application.

## 2. System Description

Nowadays, there is a great interest in using high resolution radar [11], because targets can not only be detected, but can also be classified and identified. To achieve high resolution, it is necessary to transmit a large bandwidth. The range resolution is inversely proportional to the RF transmitted bandwidth. The system works in the millimeter-wave band. Thus, a large bandwidth can be more easily transmitted because the relative bandwidth is low.

The peak power provided by commercial amplifiers in the millimeter-wave band is low. The proposed sensor has a Continuous Wave (CW) configuration in order to increase the mean power and thus the maximum range [12].

The radar transmits a Linear Frequency Modulated (LFM) signal. LFM systems obtain range information from beat frequencies. The receiver has a homodyne structure that implements a matched receiver based on correlations. LFM-CW signal facilitates system miniaturization and requires low power operation, which makes it possible to install the system in an UAV.

### 2.1. General scheme

**Figure 1.**
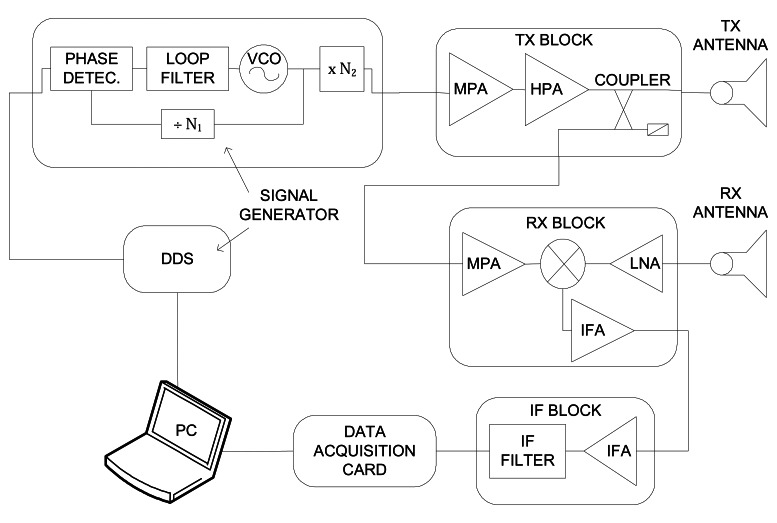
SAR system block diagram.

**Figure 2.**
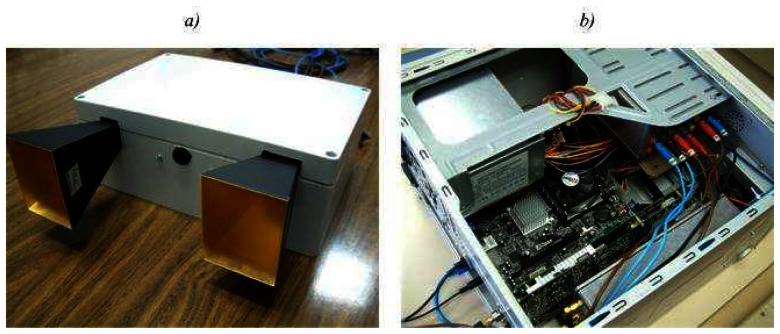
Sensor pictures a) RF subsystem b) Control subsystem.

Figure 1 illustrates the block diagram, while Figure 2 presents several pictures of the system. The RF sensor dimensions are 24×16×9 cm, and its weight is 2.5 Kg.

The received signals are down-converted to base-band mixing the echoes from the targets with a coupled sample of the transmitted signal (homodyne architecture). The isolation between the transmitter and receiver is achieved using two separate antennas for transmission and reception, which are standard rectangular horns with a beamwidth of 6° and 8°, and a gain of 24 dB (Figure 2a).

The signal generation is obtained with a closed-loop scheme. A Phase Locked Loop (PLL) generates the tuning signal to a Voltage Controlled Oscillator (VCO) using as a reference a strictly linear frequency ramp obtained thanks to a Direct Digital Synthesizer (DDS), which is controlled by a Personal Computer (PC). This PC also has an acquisition board to sample the beating frequencies, and to store them. The SAR signal processing is carried out by this PC (Figure 2b).

The most important operation characteristics are summarized in Table 1: central frequency, *f_c_*, 34 GHz (λ= 8.8 mm), and the maximum transmitted bandwidth (B_RF_), 2 GHz. The system transmits a continuous train of linear frequency ramps with a maximum Pulse Repetition Frequency (PRF) of 5 KHz. To avoid aliasing in the Doppler spectrum the maximum azimuthal sampling interval is determined to be 2.1 cm.

### 2.2. Signal generation subsystem

The signal generation subsystem consists of a PLL that compares the output signal of the VCO with a strictly linear frequency modulated signal generated by a DDS. The VCO frequency, around 8 GHz, is divided with a N_1_ divider (N_1_=80) to compare it in the PLL. The LFM-CW linear ramp of the VCO is multiplied with a N_2_ multiplier factor (N_2_=4) to obtain the desired transmitting millimeter-wave signal.

**Figure 3.**
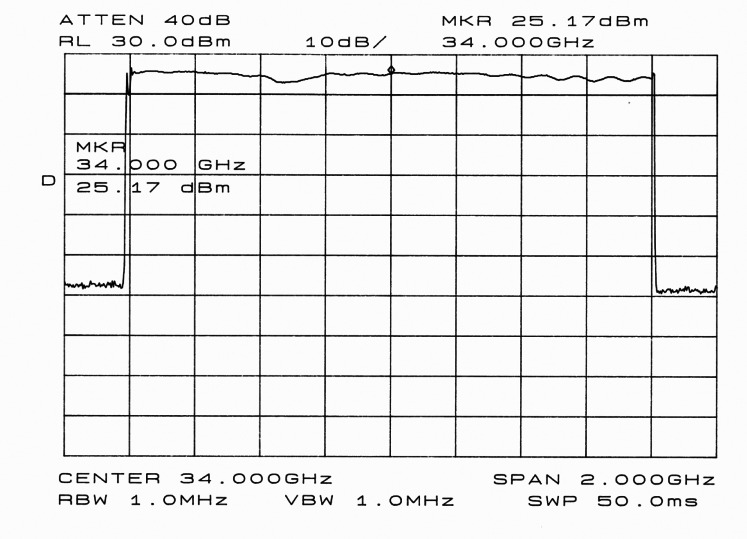
LFM-CW (*f_c_*=34 GHz, *B_RF_*=1.6 GHz) measured in the transmitter subsystem.

Figure 3 shows the measurement of a 1.6 GHz bandwidth and 34 GHz carrier frequency signal, which was measured with a 4 dB loss cable. In this case, the LFM generated with the DDS has a central frequency of 106.25 MHz and a bandwidth of 5 MHz.

### 2.3. Transmitter subsystem

The transmitter (TX) block is composed of a Medium Power Amplifier (MPA), a High Power Amplifier (HPA) and a 30 dB coupler. The measured gain of the block was around 30 dB, and the 1 dB compression point, P_1dB_, is 31 dBm.

### 2.4. Receiver subsystem

The coupled sample of the TX block, using the 30 dB coupler, was amplified with a MPA and used as the Local Oscillator (LO) signal in the receiver. The RF echoes were amplified with a Low Noise Amplifier (LNA) before mixing them with the LO. The base-band signal was amplified 20 dB before filtering it. The noise figure of the receiver is 5.2 dB.

### 2.5. IF subsystem and acquisition subsystem

The Intermediate Frequency (IF) block consists of an amplifier and a band-pass IF filter. This filter must be designed for a concrete application and is used to reduce the IF bandwidth to be sampled, and to improve the Signal to Noise Ratio (SNR).

There are several ways to tune the range interval to be acquired. In other works [13], a heterodyne receiver with fixed IF frequency and a variable mixer was used. In our sensor, a fixed central frequency IF filter and a variable PRF were used to tune the different range limits. The technique is explained in detail in [3, 4] but a short summary is given here.

The received signal from a target is demodulated with a coupled version of the transmitted signal to produce a beat signal, with frequency *f_b_*:

(1) $$f_{b}=\frac{2R}{c}\gamma \approx \frac{2R}{c}\sim B_{RF}\cdot \text{PRF}$$

The beat frequency is directly proportional to the target range *R*. A fixed IF bandwidth *f_L_* < |*f*| < *f_H_* allows collecting data from a range interval *R_min_* < *R* < *R_max_*:

(2) $$\begin{array}{cc} R_{\min}=\frac{c}{2B_{RF}\cdot \text{PRF}}f_{L}, & R_{\max}=\frac{c}{2B_{RF}\cdot \text{PRF}}f_{H} \end{array}$$

The system can be tuned to different range intervals varying the PRF with the advantage of using a same IF filter. Also, the system can make a band-pass sampling. The band-pass theorem [14] states that a band-pass signal that has a nonzero spectrum only over the frequency interval *f_L_* < |*f*| < *f_H_* can be reproduced from sampled values if the sampling frequency *f_s_* satisfies the relationships:

(3) $$\frac{2f_{H}}{n}\leq \begin{array}{cc} f_{s}\leq \frac{2f_{L}}{\left(n-1\right)}, & 2\leq n\leq \frac{f_{H}}{f_{H}-f_{L}}\text{with}\;n\in Z \end{array}$$

The sampling rate reduction allows use of a cheaper Analogue to Digital Converter (ADC), and reduces the memory and CPU requirements.

## 3. LFM-CW SAR signal processing chain

The radar receives a delayed echo of the transmitted signal, Equation (4), for each scatter in the scene. This signal is given by Equation (5), where *N_s_* is the number of scatters in the scene, and *R*(*k*,*t̂*) the range to the scatter *k^th^*. This range has two components: the range *R_i_*(*k*,*t̂*) due to the ideal trajectory of the platform and the range *R_err_* (*k*,*t̂*) due to the movement errors of the platform.

(4) $$s_{tx}\left(\hat{t},t\right)=\text{rect}\left(\frac{t}{\text{PRI}}\right)\exp\left[j\left(\omega_{c}t+\pi \gamma t^{2}\right)\right]$$

where *c* is the speed of light, *ω_c_* is the carrier pulsation, *PRI*=*1/PRF, γ*≈*B_RF_/PRI, t* is the fast time and *t̂* is the slow time.

(5) $$s_{rx}\left(\hat{t},t\right)=\sum_{k=1}^{N_{s}}a\left(k,\hat{t}\right)\cdot \text{rect}\left(\frac{t-t_{R}\left(k,\hat{t}\right)}{\text{PRI}}\right)\exp\left[j\left(\omega_{c}\left(t-t_{R}\left(k,\hat{t}\right)\right)+\pi \gamma {\left(t-t_{R}\left(k,\hat{t}\right)\right)}^{2}\right)\right]$$

where $t_{R}\left(k,\hat{t}\right)=\frac{2R\left(k,\hat{t}\right)}{c}=\frac{2\left(R_{i}\left(k,\hat{t}\right)+R_{\text{err}}\left(k,\hat{t}\right)\right)}{c}$, and *a*(*k*,*t̂*) is the backscattering coefficient of the *k^th^* scatter.

The received signal, Equation (5), is demodulated with the complex conjugate of the transmitted signal, Equation (4). The result of this operation is the signal given by Equation (6), which is band-pass filtered and sampled, resulting the digital signal shown in Equation (7).

(6) $$s_{d}\left(\hat{t},t\right)=\sum_{k=1}^{N_{s}}a\left(k,\hat{t}\right)\cdot \text{rect}\left(\frac{t-t_{R}\left(k,\hat{t}\right)}{\text{PRI}}\right)\exp\left[-j\left(\omega_{c}t_{R}\left(k,\hat{t}\right)+2\pi \gamma t_{R}\left(k,\hat{t}\right)t-\pi \gamma {t_{R}}^{2}\left(k,\hat{t}\right)\right)\right]$$

(7) $$s_{s}\left(m,n\right)=\sum_{k=1}^{N_{s}}a\left(k,m\right)\cdot \text{rect}\left(\frac{nT_{s1}-t_{R}\left(k,m\right)}{\text{PRI}}\right)\times \exp\left[-j\left(\omega_{c}t_{R}\left(k,m\right)+2\pi \gamma t_{R}\left(k,m\right)nT_{s1}-\pi \gamma {t_{R}}^{2}\left(k,m\right)\right)\right]$$

where the fast time is sampled with a sampling period *T_s_*_1_, (*t* = *nT_s_*_1_), and the slow time is sampled at *PRF* rate, *(t̂* = *m* · *PRI)*, and *m* and *n* are integer indexes with values *m*=0,…,*M*-1 and *n*=0,…,*N*-1.

After sampling, the signal given by Equation (7) was demodulated with a digital reference signal, Equation (8), and low-pass filtered. This reference signal is the echo that would have been received from a target at the centre range of the swath (range *R_0_*).

(8) $$\begin{array}{ccc} s_{\text{ref}}\left(m,n\right)=\exp\left(j\left(2\pi \gamma t_{0}nT_{s1}+\omega_{c}t_{0}-\pi \gamma {t_{0}}^{2}\right)\right), & \text{where} & t_{0}=\frac{2R_{0}}{c} \end{array}$$

This operation has two consequences. First, the band-pass signal is converted to a low-pass signal. Second, the reference signal makes motion compensation to a line, which is necessary to use RMA as the Image Formation Process (IFP) [7, 15]. At this point, the signal is a low-pass signal that can be resampled to fulfill the Nyquist sampling rate. This resampling, with a period *T_s2_*, reduces the number of samples and therefore the computational burden.

The resampled signal, Equation (9), still has three error terms: a phase error Φ*_err_* (*m*), a frequency shift (equivalent to a range shift) and the known Residual Video Phase (RVP) [7].

(9) $$\begin{array}{l} s_{\text{resam}}\left(m,n\right)=\sum_{k=1}^{N_{s}}a\left(k,m\right)\cdot \text{rect}\left(\frac{nT_{s2}-t_{R}\left(k,m\right)}{\text{PRI}}\right)\times \\ \times \exp\left[-j\left(\begin{array}{c} \frac{4\pi \gamma }{c}\left(\frac{f_{c}}{\gamma }+nT_{s2}-t_{0}\right)\left(R_{i}\left(k,m\right)-R_{0}\right)+\underset{\text{RVP}}{\underset{︸}{\frac{4\pi \gamma }{c^{2}}{\left(R\left(k,m\right)-R_{0}\right)}^{2}}}+\\ +\underset{\Phi_{\text{err}}\left(m\right)}{\underset{︸}{\frac{4\pi \gamma }{c}\left(\frac{f_{c}}{\gamma }-t_{0}\right)R_{\text{err}}\left(k,m\right)}}+\underset{\begin{array}{cc} \text{Range} & \text{shift} \end{array}}{\underset{︸}{\frac{4\pi \gamma }{c}R_{\text{err}}\left(k,m\right)\cdot nT_{s2}}}+ \end{array}\right)\right] \end{array}$$

The phase error Φ*_err_*(*m*) and the range shift were estimated and corrected using an autofocus technique. PGA, which has been extended to stripmap mode, is the chosen algorithm to estimate and correct the motion errors. The implemented algorithm is explained in the next section.

The RVP term can be removed by means of the technique known as range deskew [7].

After these corrections, the signal given by Equation (10) is obtained. This is the signal desired by RMA to obtain a perfectly focused SAR image [7].

(10) $$s_{\text{corr}}\left(m,n\right)=\sum_{k=1}^{N_{s}}a\left(k,m\right)\cdot \text{rect}\left(\frac{nT_{s2}-t_{0}}{\text{PRI}}\right)\exp\left(-j\left(\frac{4\pi \gamma }{c}\left(\frac{f_{c}}{\gamma }+nT_{s2}-t_{0}\right)\left(R_{i}\left(k,m\right)-R_{0}\right)\right)\right)$$

## 4. Motion error compensation

### 4.1. Phase Gradient Autofocus (PGA)

This subsection only shows an overview of the PGA algorithm. The detailed description can be found in [5, 6, 7]. PGA is able to estimate and remove arbitrary phase errors in SAR images. The right operation of spotlight PGA is based on three hypotheses:

- The phase error must be spatially invariant over the entire image, that is, all the scatters in a scene must share the same phase error history.
- Targets must exist with phase error information over the entire data set.
- The raw data signal must be related to the SAR image by means of a two-dimensional Fourier Transform. This condition is always fulfilled in spotlight SAR, because the data are usually motion compensated to a point. However, our radar is going to operate at stripmap-mode to maximize the coverage of the scene.

The next four points summarize spotlight PGA:

1. Circular shifting: The input to PGA is the complex SAR image. The first step is to choose the strongest target for each range bin and circularly shift it to the azimuthal origin. This shift removes the Doppler frequency offset of these scatters. Therefore, PGA can not estimate linear phase errors in the Doppler history.
2. Windowing: Each selected scatter is windowed azimuthally. Windowing isolates the phase error information contained at the dominant scatter from other scatters at the same range bin that could interfere with the phase error estimation.
3. Phase gradient estimation: The circular shifted and windowed data are inversely Fourier transformed along azimuth to the range-compressed domain. Let us denote *S(m,n)* as the signal associated with the windowed target from the *n^th^* range bin, i.e., the windowed and shifted Fourier transform across the columns of *s(m,n*):
  (11) $$S\left(m,n\right)=\left|S\left(m,n\right)\right|\exp\left(j\left[\Phi_{\text{err}}\left(m\right)+\theta \left(m,n\right)\right]\right)$$
  where Φ*_err_*(*m*) is the phase error (spatially invariant) and *θ*(*m*,*n*) is the scatter-dependent phase function for the chosen target in the *n^th^* range bin. The linear unbiased minimum variance estimate of the gradient phase error is given by:
  (12) 〈Φerr′(m)〉lumv=∑nIm{S*(m,n)S′(m,n)}∑n|S(m,n)|2
  where *S*′(*m*,*n*) is the first derivative along columns of *S(m,n)*.
  There are other phase gradient estimators that produce good results in combination with circular shifting and windowing [5, 7, 16].
4. Iterative phase correction: The estimated phase gradient has to be integrated to obtain the phase error. Any constant or linear term of the phase error is removed. After the integration, the phase error is corrected multiplying the range-compressed data with a complex exponential function whose phase is the inverse of the phase error estimate. Then the corrected data are Fourier transformed to the image domain, leading to a more focused image. All the described steps are iteratively repeated until some convergence criterion is reached [5, 7].

Basic spotlight PGA is based on the three fundamental hypotheses that have been explained above. The proposed SAR signal processing chain implements a stripmap-mode with motion compensation to a line. The first difference is referred to the azimuthal resolution limit and the scene coverage. In stripmap-mode, the antenna beamwidth limits the azimuthal resolution, and data collection length determines azimuthal scene size. However, in spotlight-mode, data collection length determines azimuthal resolution and antenna beamwidth limits the azimuthal scene size.

Both modes usually use the same transmitted signal and a dechirp-on receive procedure [7]. However, the second difference is the reference signal used for dechirping. Stripmap-mode uses compensation to a line that preserves the azimuthal chirp, while spotlight-mode uses motion compensation to a point that removes this azimuthal chirp [7].

The proposed stripmap PGA implements different strategies to fulfill these PGA hypotheses. The implemented algorithm is described in the next subsections. The movement error that has been estimated by PGA is used in a two step correction method: a coarse correction that removes the range shift, and a fine correction that focuses the target within the resolution cell.

### 4.2. Stripmap to spotlight conversion

Spotlight PGA needs a raw signal data that can be compressed by means of a two dimensional Fourier transform to form a SAR image. However, stripmap data preserve the azimuthal chirp and a linear range migration, that is, a single scatter is detected at different range bins for each pulse of the data set. These stripmap characteristics do not allow compression of the image using a two-dimensional Fourier transform. To solve this problem, the proposed algorithm converts stripmap data to spotlight data. The conversion consists of a change of the reference signal. Stripmap data have been motion compensated with a fixed reference range *R_0_*, while spotlight data were motion compensated with a different reference range *R_c_(m)* for each pulse. The stripmap to spotlight conversion removes the stripmap motion compensation and applies motion compensation to the scene center. This is equivalent to multiply each pulse with a complex exponential given by equation. (13):

(13) $$s_{st2sp}\left(m,n\right)=\exp\left[j\left(\frac{4\pi }{\lambda }\left(R_{0}-R_{c}\left(m\right)\right)+\frac{4\pi \gamma }{c}\left(R_{0}-R_{c}\left(m\right)\right)\cdot n\cdot T_{s2}+\frac{4\pi \gamma }{c^{2}}\left({R_{c}}^{2}\left(m\right)-{R_{0}}^{2}\right)\right)\right]$$

where *λ* is the wavelength.

This conversion removes the azimuthal chirp without modifying the phase error that must be estimated [7]. PGA needs targets with phase error information over the entire data collection range. In spotlight-mode, the phase history of any point covers the entire data set. However, in stripmap-mode the phase history of any scatter is limited by the antenna beamwidth and is contained only in a portion of the data set. A solution was proposed in [17]. It consists of dividing the complete data collection into azimuthal data segments. Our proposed PGA uses this philosophy and independently converts each segment from stripmap to spotlight mode. Furthermore, this minimizes range migration even in the case of squinted stripmap data.

Both spotlight and stripmap data with dechirp-on receive have a RVP term. In both modes, RVP can be removed using the range deskew filter given by Equation (14) [7].

(14) $$H_{rd}\left(f\right)=\exp\left(-j\frac{\pi }{\gamma }f^{2}\right)$$

### 4.3. Range alignment previous to the phase estimation

For each azimuthal data segment, some prominent scatters must be selected to estimate the phase error due to the non-ideal movement. This selection can be done in the range compressed domain. Several methods to choose prominent scatters in the range compressed domain have been proposed in the specialized literature about ISAR [19, 20]. For our purpose, the most appropriate algorithm is based on the minimal normalized variance criteria. It computes the normalized variance for each range bin (column of the range compressed map). The range bin *n̂* with the minimal normalized variance is chosen. The formulation is given by Equation (15):

(15) $$\begin{array}{ccc} \hat{n}=\arg_{n}\min[V\left(n\right)] & \text{with}V\left(n\right)=\frac{{\overset{M-1}{\underset{m=0}{\text{∑}}}\left[\left|S\left(m,n\right)\right|-\bar{S}\left(n\right)\right]}^{2}}{\overset{M-1}{\underset{m=0}{\text{∑}}}{\left|S\left(m,n\right)\right|}^{2}} & \text{and}\;\bar{S}\left(n\right)=\frac{1}{M}\overset{M-1}{\underset{m=0}{\text{∑}}}\left|S\left(m,n\right)\right| \end{array}$$

where *S(m,n)* is the range compressed map, with *M* azimuthal cells (rows), and *N* range bins (columns).

In this way, we achieve selected targets that are located in an azimuthal coordinate quite centered respect to the middle of the azimuthal data segment. Taking short enough azimuthal data segments, we can avoid the linear range migration due to the ideal motion of the platform. It means that a selected target has remained in the same range cell for the major part of the azimuthal data segment and, therefore, this range column is the most appropriate to extract the movement error.

Unfortunately, the range migration cell due to non-ideal motion is still present in the range compressed domain. The selected range column could be processed by the typical PGA to extract the movement error. However, the range column does not have information in zones where the movement error was larger than the range resolution, because the target has suffered a range shift. One way to overcome this drawback is range aligning the history of the target in the range compressed domain. There are several ISAR techniques that make this correction. Also, we have to take into account that this range alignment is usually the first step for motion compensation in ISAR. For example, EC [8, 9] or GRA [10] are robust techniques for aligning the history of a target in the same range column in presence of noise and clutter.

**Figure 4.**
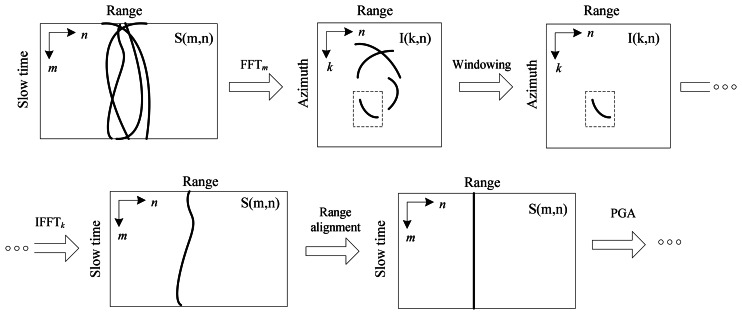
Range alignment procedure.

To avoid the interference of other targets in the range compressed domain, we can compress the image azimuthally via a Fourier transform and isolate the selected prominent scatter in the image domain using a window. This technique is used in ISAR processing [21]. The complete procedure is illustrated in Figure 4.

After the range alignment, the column can be processed by the basic PGA to estimate the phase error due to non-ideal motion.

### 4.4. Phase gradient estimation

Only the scatters that are illuminated during all the acquisition time have complete phase error information. The geometry of stripmap-mode does not allow collection of data from the same scatter all the time. Returns from a target are only received for a limited number of pulses. The number of received pulses is determined by the antenna beamwidth. This is a drawback, because PGA needs targets with phase error information over the entire data set.

The solution proposed in [17] consists of dividing the data set into shorter azimuthal data segments. After this division, each azimuthal data segment is independently converted from stripmap to spotlight-mode in the manner described above. Each segment is range compressed via a Fourier transform. For each segment, several columns containing prominent targets are selected using minimal normalized variance. The history of these targets is range aligned as previously explained. Then, basic spotlight PGA is independently applied to each column to obtain a phase error estimate. Final phase error of the segment will be an average of these independent estimations.

It is known that PGA can not estimate linear phase errors. Thus, the phase error estimation of each segment could exhibit a different linear component. To avoid discontinuities in the phase error estimation between consecutive segments, in [17] it is proposed to remove the mean of the phase gradient before combining and integrating the phase error segments. However, this procedure leads to large errors because the segments are small, and the linear component of the actual phase error is, in fact, different from zero and also different from one segment to another.

An estimate of the second derivative of the phase error has the advantage of forcing the mathematical continuity between consecutive segment estimations. The second derivative estimate of the phase error from all the segments is integrated together yielding the phase error gradient without any discontinuity over the complete data set. The mean of the whole phase gradient was then removed before integrating again to get the complete phase error.

The first hypothesis of PGA is that the phase error must be spatially invariant over the entire image, i.e. all the scatters in a scene must share the same phase error. Traditional PGA algorithm assumes that the phase error is constant with range. However, with wide swaths or short ranges, the incidence angle is highly varied and the phase error due to the movement exhibits range dependence. Two alternatives have been proposed:

The first one consists of dividing the data set into narrow range subswaths. The subswath width has to be chosen to guarantee high correlation of the phase error within the subswath. The division is carried out with the range compressed data, after azimuthal segmentation and stripmap to spotlight conversion. The previously described PGA procedure is independently applied to each subswath. The selected targets of one subswath will have very similar phase errors, and the PGA correction will be appropriate to all the targets within the subswath.

The second alternative is to estimate the range dependence of the error to correlate different subswaths. This method is based on the Phase Weighted Estimation PGA (PWE-PGA) described in [18]. This method improves the performance of PGA, but increases its computational complexity.

### 4.5. Coarse movement correction

Once the phase error due to non-ideal motion has been estimated, the movement error can be extracted using the phase information. Usually *t*_0_ is negligible in comparison with $\overset{f_{c}}{\;}/\underset{\gamma }{\;}$ and, therefore, *R_err_*(*m*) can be approximated by:

(16) $$R_{\text{err}}\left(m\right)\approx -\frac{\lambda }{4\pi }\Phi_{\text{err}}\left(m\right)$$

where Φ*_err_*(*m*) is the estimated phase error and *R_err_*(*m*) is the estimated motion error on the slant plane.

This estimate is valid to correct the range shifts due to the movement errors. The range shift can be removed using the original stripmap raw data. The procedure consists of shifting each received pulse the quantity given by *R_err_*(*m*). The shift can be performed by multiplying each *m^th^* pulse of the raw matrix *s(m,n)* with a complex exponential given by (17).

(17) $$g_{\text{coarse}}\left(m,n\right)=\exp\left(j\cdot \frac{4\pi \gamma }{c}R_{\text{err}}\left(m\right)\cdot nT_{s2}\right)$$

After this correction, the movement errors that are larger than the resolution cell have been compensated, i.e. the range shift due to non-ideal motion has been removed. However, the range curvature due to ideal motion that is different for each scatter still remains. This range curvature will be compensated using RMA [7, 15].

The range error estimated by (16) is extracted from the phase information. This range information is only valid if the estimated phase error can be unwrapped, i.e. if the phase difference between two consecutive pulses is lower than *π* radians:

(18) $$\frac{4\pi }{\lambda }\left|R_{\text{err}}\left(m\right)-R_{\text{err}}\left(m-1\right)\right|<\pi$$

Equation (18) imposes a limit for the maximum instantaneous radial velocity error (projected over slant plane velocity) that can be corrected. Using Equation (19), for our system the instantaneous maximum radial velocity error will be 11 m/s, which is a high value.

(19) $$\left|v_{\text{slant}}\right|<\frac{\lambda }{4}\text{PRF}$$

### 4.6. Fine movement correction

The coarse correction removes the range shift, but a fine correction is still necessary, i.e. the typical phase correction of PGA. Once the phase error component has been estimated and the range shift removed, the corrected stripmap data set can be Fourier transformed to the range domain, and the phase error estimation can be removed for each range bin. Let *S(m,n)* be the range compressed data of *s(m,n)*. The correction consists of multiplying each column (range bin) of the matrix *S(m,n)* with a complex exponential given by Equation (20):

(20) $$G_{\text{fine}}\left(m\right)=\exp\left(j\cdot \Phi_{\text{err}}\left(m\right)\right)$$

Finally, the range compressed data are inversely Fourier transformed again to the time domain. This data matrix contains the stripmap signal in the format required by RMA, without motion error and without RVP.

### 4.7. Movement error analysis

**Figure 5.**
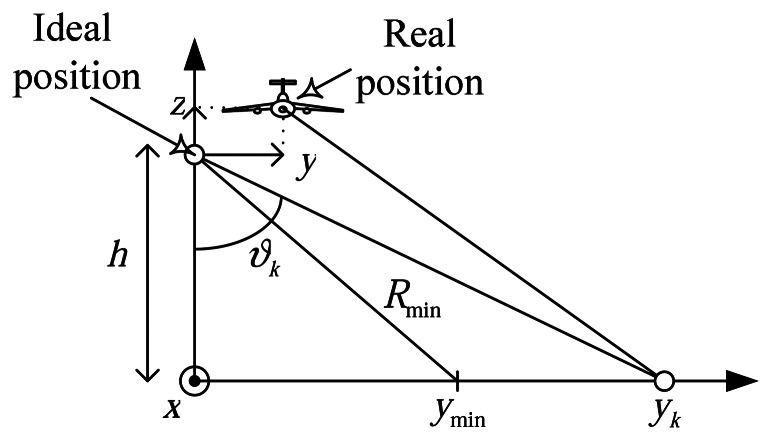
Range dependant error due to non-ideal motion.

Let there be a trajectory error with **z** and **y** direction components, see Figure 5. These movements, *y(m)* and *z(m)*, induce a phase error and a range shift on the slant plane that depends on the range to the targets *y_k_*. The proposed algorithm can correct range dependencies due to non-planar movement of the platform.

The phase error can be written as:

(21) $$\Phi_{\text{err},k}\left(m\right)=\frac{-4\pi }{\lambda }[y\left(m\right)\cdot \sin\vartheta_{k}-z\left(m\right)\cdot \cos\vartheta_{k}]=\Phi_{y}\left(m\right)\cdot \sin\vartheta_{k}+\Phi_{z}\left(m\right)\cdot \cos\vartheta_{k}$$

For a target located in the center of the *k^th^* range bin the incidence angle *ϑ_k_* is:

(22) $$\vartheta_{k}=\cos^{-1}\left(\frac{h}{R_{\min}+k\cdot \delta R}\right)$$

where *δR* is the range resolution of the image

The Φ*_y_*(*m*) and Φ*_z_*(*m*) phase error components do not depend on *ϑ_k_* and, therefore, they are the same for all the range bins. This is exploited by PWE-PGA, described in [18]. Therefore, this non-ideal motion can be compensated by the proposed autofocus algorithm described above.

Variations in along-track speed result in non-uniform spacing of the radar pulses. This non-uniform sampling of the Doppler spectrum results in erroneous calculations of the Doppler phase history. Traditionally, the data are interpolated in the azimuthal direction to correct the velocity variations across the synthetic aperture [2]. This interpolation is based on the measured data of an IMU. Our system does not have an available IMU, therefore, this kind of error can not be compensated. However, our autofocus is able to correct constant deviations respect to the nominal velocity, that is, the mean velocity error.

A constant deviation of the speed generates a phase error that produces defocus and distortion in the image. An analytic study of this phase error can be obtained with a simple geometrical development.

Suppose a target located on the ground in a position with coordinates (*x_p_*, *y_p_*). The phase of the received signal for each pulse is directly proportional to the distance between the platform and the target *R_p_*(*t̂*):

(23) $$\Phi \left(\hat{t}\right)=-\frac{4\pi }{\lambda }\cdot R_{p}\left(\hat{t}\right)$$

where *t̂* is the slow time and *λ* is the wavelength of the transmitted signal.

The range between the platform and the target varies for each pulse if the platform moves at different speed from the nominal one. Suppose a nominal velocity of the platform, *V_a_*, this range is given by:

(24) $$R_{p}\left(\hat{t}\right)=\sqrt{{r_{p}}^{2}+{\left(x_{p}-V_{a}\cdot \hat{t}\right)}^{2}}$$

where $r_{p}=\sqrt{h^{2}+{y_{p}}^{2}}$ is the distance between platform and target projected over the **y-z** plane.

Supposing *x_p_* and *V_a_* · *t̂* < *r_p_*, Equation (24) can be approximated by:

(25) $$R_{p}\left(\hat{t}\right)=r_{p}+\frac{{\left(x_{p}-V_{a}\cdot \hat{t}\right)}^{2}}{2\cdot r_{p}}$$

Now, suppose that the platform moves with a constant deviation velocity, Δ*V_a_*, over the nominal speed *V_a_*. The new range between the platform and the target, *R_bias_*(*t̂*), is given by:

(26) $$R_{\text{bias}}\left(\hat{t}\right)=\sqrt{{r_{p}}^{2}+{\left(x_{p}-\left(V_{a}+\Delta V_{a}\right)\cdot \hat{t}\right)}^{2}}$$

Supposing *x_p_* and (*V_a_* + Δ*V_a_*)·*t̂* ≪ *r_p_*, Equation (26) can be approximated by:

(27) $$R_{\text{bias}}\left(\hat{t}\right)=r_{p}+\frac{{\left(x_{p}-\left(V_{a}+\Delta V_{a}\right)\cdot \hat{t}\right)}^{2}}{2\cdot r_{p}}$$

Using Equation (23) that relates range with phase, the phase error due to a non-zero mean velocity error is given by:

(28) $$\Phi_{\text{err}}\left(\hat{t}\right)=-\frac{4\pi }{\lambda }\cdot \left(R_{\text{bias}}\left(\hat{t}\right)-R_{p}\left(\hat{t}\right)\right)$$

Developing:

(29) $$\Phi_{\text{err}}\left(\hat{t}\right)=-\frac{2\pi }{\lambda }\cdot \frac{\Delta V_{a}\cdot \left(\Delta V_{a}+2\cdot V_{a}\right)}{r_{p}}\cdot {\hat{t}}^{2}+\frac{4\pi }{\lambda }\cdot \frac{x_{p}\cdot \Delta V_{a}}{r_{p}}\cdot \hat{t}$$

The phase error consists of two different components. The first term is a quadratic error. This error produces a loss of resolution. The second term is a linear error which produces an azimuthal shift of the image.

The quadratic error depends on the projected distance over the **y-z** plane, *r_p_*. Therefore, this error is different for each range bin, because it depends on the position of the target in **z** and **y**. However, this kind of error can be compensated by the autofocus, because it perfectly matches with the error model of the method PWE-PGA described in [18].

The linear phase error cannot be corrected. It is space variant because depends on the projected distance over the **y-z** plane, *r_p_*, and the azimuthal coordinate of the target *x_p_*. Therefore, this error distorts the image. The permitted distortion gives the maximum allowed velocity deviation, Δ*V_a_*.

### 4.8. Simulated results

The algorithm was tested with simulated stripmap-mode data. The simulated system has the same parameters as our real LFM-CW SAR system. The nominal resolution cell chosen for this experiment is 15 cm (24 cm with windowing). The simulation evaluated the degradation of the impulse response of the SAR system. The measured parameters were azimuth and slant range resolution at -3 dB and -9 dB respect to the main lobe level, and azimuth and slant range PSLR (Peak Side-Lobe Ratio) with respect to the main lobe level.

**Figure 6.**
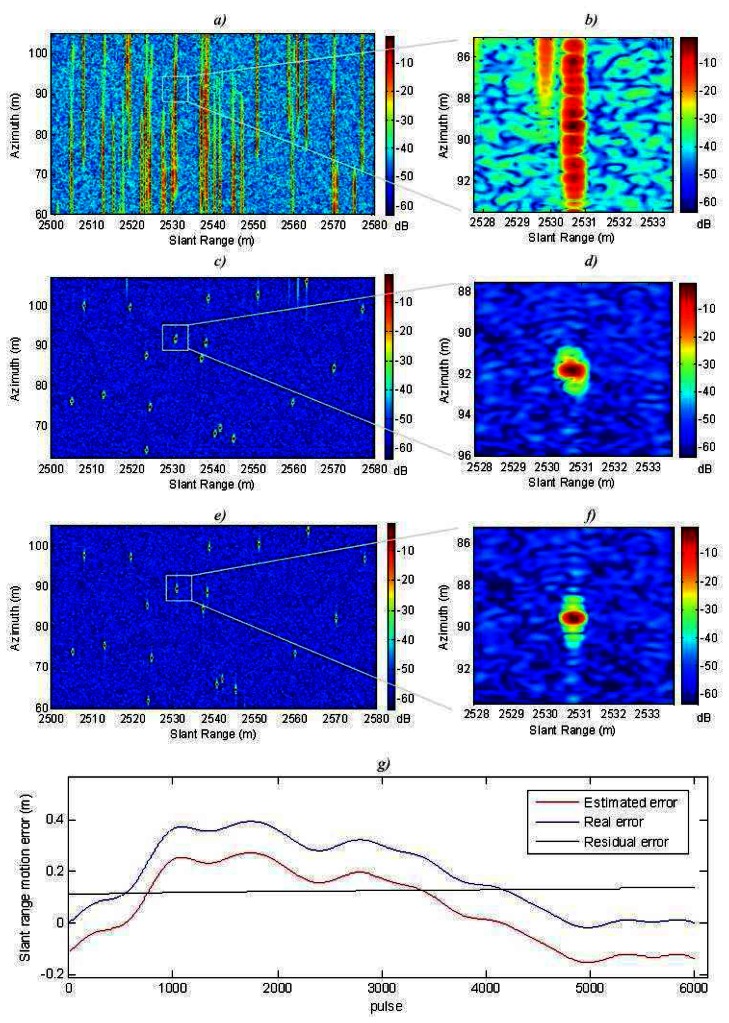
Simulated motion compensation: a) SAR image without motion compensation; b) Detail of a target without motion compensation; c) SAR image only with fine correction; d) Detail of a target with only fine correction; e) SAR image with coarse and fine correction; f) Detail of a target with coarse and fine correction; g) Movement errors.

The simulated scene consists of several corner reflectors uniformly distributed in a swath of 45×80 m. The swath is 2500 m away from the radar. The movement error consists of random shifts in the **y-z** plane, see Figure 5. The combination of these errors generated a range shift in the slant plane larger than the range resolution. This motion error is shown in Figure 6g. Also, the estimated motion error by the autofocus is shown in Figure 6g. Our algorithm appears to have good accuracy. The residual error is linear and can not be compensated by the autofocus. However, it is not important because these errors do not result in image defocusing [7].

Figure 6a shows the SAR image and Figure 6b shows the detail of the system impulse response without applying any autofocus technique. The image is completely defocused. Figure 6c illustrates a more focused SAR image. This is the result of applying the autofocus only with fine correction, without coarse correction. The detail of the system impulse response can be seen in Figure 6d. Figure 6e and 6f respectively show the SAR image and the detailed system impulse response applying the complete autofocus. Thanks to the novel coarse compensation we can appreciate a resolution improvement of 7 % in slant range, and 11 % in azimuth. Also a slight increment of the PSLR is achieved. Table 2 gives the exact measured values.

## 5. Real data using a ground mobile platform

**Figure 7.**
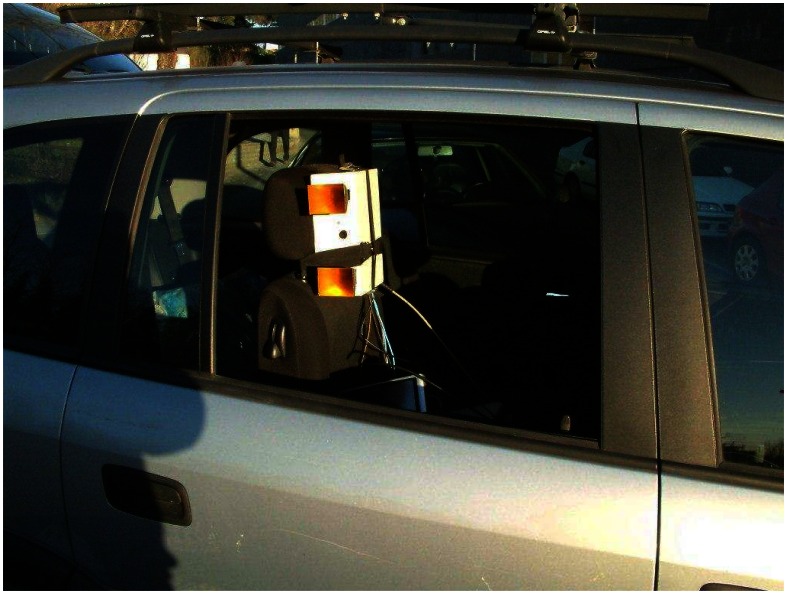
Automobile SAR system.

The LFM-CW radar has been installed in a car that has been used as mobile platform, see Figure 7. Two real experiments have been carried out to evaluate the performance of our radar as sensor of a SAR system. The nominal square resolution cell is 30 cm.

**Figure 8.**
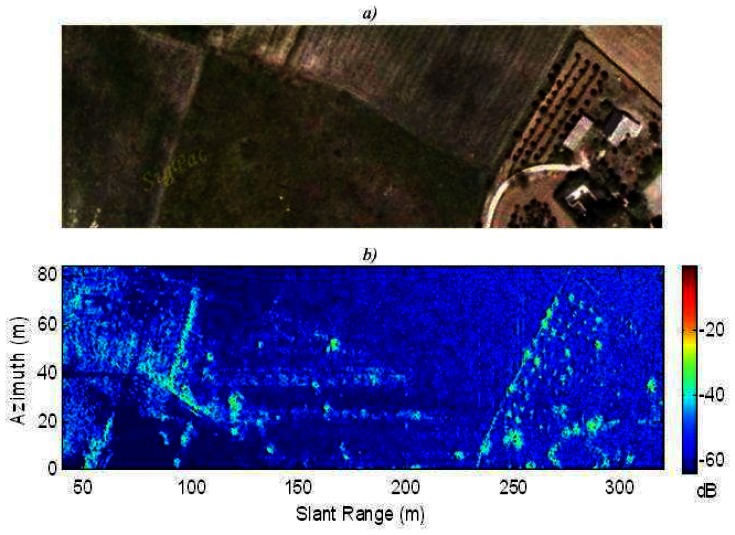
Area with man-made objects and vegetation a) Optical image b) High resolution SAR image.

The first experiment consists of obtaining a high resolution SAR image of a zone with man-made objects and vegetation. Figure 8 illustrates the SAR image that has been achieved and an optical image to compare. Most of the objects can be detected thanks to the high resolution. We can observe that many shadowing effects are presented because the system was being operating at ground level. Furthermore, the noise level was high, but it is interesting to remark that the incidence angle was near to 90 degrees, that is, the waves were almost tangent to the terrain surface. Therefore, the backscattering from the scene was very low. These two effects will not appear in UAV or airborne operations. Furthermore, this image has been processed without motion compensation because the movement errors were negligible.

**Figure 9.**
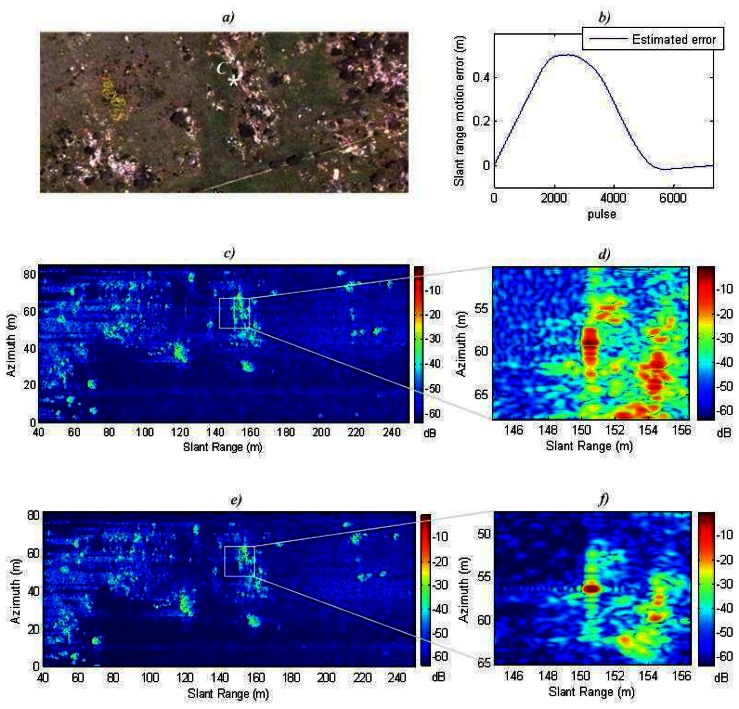
Rustic area with corner reflector *C* a) Optical image b) Estimated motion error c) SAR image without motion compensation d) Detail of the corner reflector *C* response (PSF) without motion compensation e) SAR image with motion compensation f) Detail of the corner reflector *C* response (PSF) with motion compensation.

The second experiment consisted of illuminating a rustic scene with the radar. Previously, one corner reflector was placed in the zone to measure the real impulse response of the system. This target was labeled *C* in Figure 9a that shows an optical image of the zone. The Point Spread Function (PSF) of the system can be estimated by measuring the impulse response of the system to this reflector. Figure 9a shows the optical image. Figure 9b illustrates the movement error estimated by the proposed algorithm. Figure 9c shows the SAR image of the scene without motion compensation. Also, a detail of the measured PSF without motion compensation is shown in Figure 9d. Figure 9e shows the SAR image of the scene with our motion compensation. The detail of the measured PSF with motion compensation is shown in Figure 9f. After motion compensation, the estimated PSF shows that the system has a square resolution cell around the nominal (30 cm), and a PSLR larger than 20 dB.

These two experiments have proved the feasibility and the right performance of the complete system, hardware and software, to use our miniaturized radar as a SAR sensor in UAV applications.

## 4. Conclusions

A millimeter-wave LFM-CW radar has been described. Some measurements of the subsystems have been presented. This sensor is modular, compact and lightweight, so it is very attractive for use in portable applications like UAV operation. The radar has a CW configuration to provide enough power to operate at medium range. It works in millimeter-wave band, so a large RF bandwidth is transmitted and a high range resolution is achieved.

This work is the first step towards proving the feasibility of our millimeter-wave LFM-CW radar as a SAR sensor. The sensor has been integrated in a ground SAR system using a car as mobile platform. Ground SAR systems have more shadowing effects, worse SNR and smaller coverage than airborne systems. However, two high resolution ground SAR images have been presented to demonstrate the performance of the system and the feasibility for UAV applications. Furthermore, an autofocus algorithm has been developed to correct movement errors larger than the resolution cell for UAV applications. The paper describes the algorithm and shows the obtained results.

## Figures and Tables

**Table 1.** SAR system parameters.

**Parameters**	**Values**

Central frequency *f_c_*	34 GHz
Transmitted Power *P_tx_*	30 dBm
Waveform	Chirp
Maximum RF bandwidth *B_RF_*	2 GHz
Sampling frequency *f_s_*	200 MHz
Antenna beamwidth *θ_3dB_(azimuth, elevation)*	(6 °, 8 °)
Maximum azimuthal sampling interval *Δx*	2.1 cm
Maximum PRF	5 KHz
Maximum chirp rate *γ*	10 MHz/μs

**Table 2.** SAR system impulse response measurements.

COARSE CORRECTION	Azimuth Resolution (cm)		Slant Range Resolution (cm)		Azimuth PSLR (dB)	Slant Range PSLR(dB)

	-3 dB	-9 dB	-3 dB	-9 dB		

NO	26.4	45.3	31.0	52.8	-23.8	-50
YES	24.4	41.8	27.7	46.8	-24.4	-50
